# Plasmonic enhancement of photovoltaic characteristics of organic solar cells by employing parabola nanostructures at the back of the solar cell

**DOI:** 10.1039/d3ra03637e

**Published:** 2023-09-06

**Authors:** Pankaj Kumar Das, Anuj Dhawan

**Affiliations:** a Department of Electrical Engineering, Indian Institute of Technology Delhi Hauz Khas New Delhi 110016 India adhawan@ee.iitd.ac.in

## Abstract

In this paper, we demonstrate the enhanced performance of organic solar cells (OSCs) comprising low band gap photoactive layers (PMDPP3T:PC70BM) and 2-dimensional (2D) arrays of either Ag nano-spheres, nano-hemispheres, or nano-parabolas embedded at the back of the OSCs. Finite-difference time-domain (FDTD) simulations were performed to compare the performance of the OSCs containing the different plasmonic nanostructures, in terms of optical absorption, short circuit current density (*J*_SC_) and power conversion efficiency (PCE). The results demonstrate that single junction OSCs consisting of this new active layer polymer (PMDPP3T), blended with PC70BM, and plasmonic nanostructures at the back of the OSC can enhance the optical absorption in the visible and the NIR region. We demonstrate that the aspect ratio of the nanoparticles embedded at the back of OSCs is a vital parameter for light absorption enhancement. It is observed that the performance in terms of *J*_SC_ and PCE enhancement of OSC having 2D arrays of Ag nano-parabola at the back of the solar cell improved by 26.41% and 26.37%, respectively, compared to a planar OSC. The enhancement in photon absorption can be attributed due to the enhancement of light scattering from metallic nanostructures near their localized plasmon resonance.

## Abstract 2

We demonstrate the enhanced performance of organic solar cells (OSCs) comprising low band gap photoactive layers (PMDPP3T:PC70BM) and 2-dimensional arrays of either Ag nanospheres, nano-hemispheres, or nano-parabolas embedded at the back of the OSCs.

## Graphical abstract

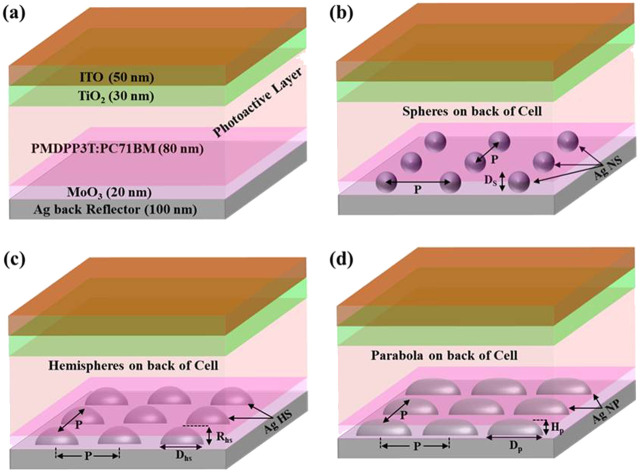

## 1. Introduction

Due to the high cost of silicon material and its processing, silicon solar cells are prohibitively expensive.^1,2^ Researchers are looking at new materials for next-generation cost-effective photovoltaics, such as thin film photovoltaics,^3–5^ dye sensitized photovoltaics,^6–8^ quantum-dot photovoltaics,^9–11^ organic photovoltaics,^12–14^ and perovskite photovoltaics.^15–17^ Despite the fact that perovskite solar cells have the best efficiency of the photovoltaics listed above ,^18,19^ their stability is low, and they tend to degrade.^20–22^ Organic solar cells (OSCs), which can be produced at a cheaper cost with earth-abundant materials^23^ and have long-term stability,^24,25^ are currently the suitable option to replace high-priced silicon photovoltaics. In addition, the unique properties of OSCs such as semi-transparency, mechanical flexibility, being light-weight and the ability to fabricate them through solution methods at room temperature make them an extremely dependable energy resource in the current photovoltaic regime.^26–31^ In the recent research work on OSCs, bulk heterojunction (BHJ) based OSCs have demonstrated good performance due to the larger interfacial contact area between the donors and the acceptors within the dimensions of the exciton diffusion length, which leads to enhanced charge separation. However, the power conversion efficiency (PCE) of BHJ based OSCs is still poor compared to silicon based solar cells. This is because of short exciton diffusion lengths *i.e.*, 10–20 nm and poor charge mobility *i.e.*, 10^−5^ to 10^0^ cm^2^ V^−1^ s^−1^ in most of the polymeric organic semiconductors.^32^ The short exciton diffusion lengths limit the active layer (AL) thickness to less than 100 nm,^33^ which inevitably decreases the photon absorption in the AL of OSC. Therefore, increasing the optical absorption in OSCs having low values of active layer thickness remains a challenge. Several light management techniques have been proposed and studied for enhancing the performances of OSCs. The inclusion of metallic nanoparticles (NPs) in the charge transport layer or in the AL of the OSCs have attracted immense interest in the past few years due to the excitation of localized surface plasmons (LSPs) in these nanoparticles.^34–37^ When nanoparticles are incorporated in the AL of the OSCs, the enhancement of the localized electromagnetic fields around the nanoparticles can contribute to some extent towards the enhancement of the absorption by the solar cell. The incorporation of metallic nanoparticles into the charge transport layer appears to be a good way to improve light absorption without hampering the interfacial morphology of donor–acceptor material in AL or quenching of photogenerated excitons, both of which could have a negative impact on device performance. But when the metallic nanoparticles incorporated in the charge transport layer are not in close proximity with the AL, it is assumed that the forward scattering of the incident light is the primary cause of absorption enhancement. Fung *et al.*^38^ demonstrated theoretically that the plasmonic enhancement of the local EM fields (due to the LSPR effect of metallic nanoparticles) incorporated in the charge transport layer does not extend into the AL of OSC.^38^ Recently, Lee *et al.* also demonstrated that the spatial extent of the light absorption enhancement near the plasmonic nanoparticle is very low, and it rapidly decreases and falls below 10% at distances of about 10 nm.^39^ It can be concluded from all these studies that forward scattering is the primary mechanism that is responsible for efficiency enhancement when the metallic nanoparticles are embedded in charge transport layer.

A significant number of studies have shown the placement of metallic NPs in the AL to increase the light absorption in the AL of OSCs.^33,36,37,40,41^ As the metallic NPs are incorporated in the AL, the near-field effect is stronger compared to the case when the metallic-nanoparticles are incorporated in the charge transport layer. Further, the metallic nanoparticles inside the AL also scatter the light within the active material of OSC, which leads to an increased light absorption. Wang *et al.* recently demonstrated ∼32% PCE improvement in OSCs containing Au NPs in the AL.^42^ Moreover, Ahn *et al.* presented theoretical as well as experimental results to show that the presence of metallic nanoparticles inside the AL leads to significantly higher field strengths in the AL, which leads to an improvement in light absorption of more than 100% at the LSPR wavelengths.^43^ When the dimensions of metal nanoparticles incorporated inside the AL are well below the wavelength of the incident light, the scattering (*C*_scatt_) and the absorption (*C*_abs_) cross-sections for a spherical nanoparticle can be estimated as:^44^1
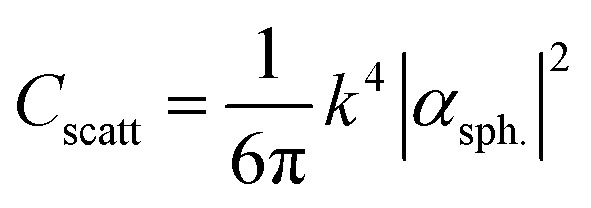

2 *C*_abs_ = *k* Im[*α*_sph._]

where *k* = 2π/*λ* denotes the wavenumber of the incident light and *α*_sph._ is the polarizability of a spherical nanoparticle, given by:3
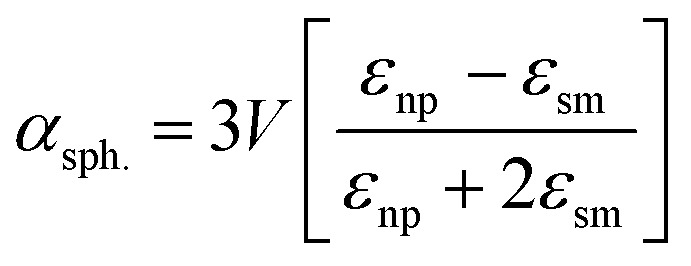
and where *V* is the volume of spherical nanoparticle, *ε*_np_ is the permittivity of the metallic nanoparticles and *ε*_sm_ is the permittivity of the surrounding medium. The scattering frequency, *Q*_scatt_ is defined as:^45^4
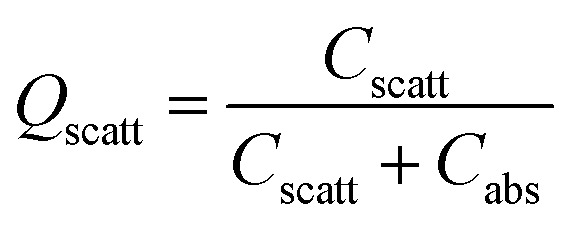


From the above equations, it can be concluded that the metallic nanoparticles embedded inside the active layers can act either as near-field enhancers or as light scattering centers or both, depending on the nanoparticle volume, geometrical shape, nanoparticle material, and the optical constants of the surrounding medium.^46^ The absorption efficiency dominates for small nanoparticles while the scattering efficiency is dominant for nanoparticles having larger dimensions. At LSPR, the scattering cross-section of large-sized nanoparticles is significantly larger than their geometric cross-section.^44^ It can therefore be concluded that particle size influences the relative significance of absorption and scattering. Thus, in the ongoing research work on OSCs, the size of the nanoparticles embedded inside the AL needs to be optimized to achieve maximum light scattering inside the AL. On the other hand, when the nanoparticles are embedded at back of solar cell are periodically arranged, the incident light can excite surface plasmon polaritons (SPPs) at the nanoparticle-AL interface.

In the regime of plasmonic research, there have been two widely used plasmonic materials are gold (Au) and silver (Ag). The Au based nanoparticles show higher absorption loss in the visible range compared to Ag based nanoparticles but have higher chemical stability. Although, both Ag and Au nanoparticles exhibits enhanced absorption and scattering efficiencies, Au NPs exhibits higher absorption than scattering. Thus, the Au NPs have a substantially lower scattering-to-absorption power ratio compared to Ag NPs. Hence, Ag NPs perform better than Au NPs in terms of scattering enhancement.^47^

In this paper, we demonstrate an OSC comprising a relatively new low band gap (LBG) semiconducting polymer, poly[[2,5-bis(2-hexyldecyl)-2,3,5,6-tetrahydro-3,6-dioxopyrrolo[3,4-*c*]pyrrole-1,4-diyl]-*alt*-[3′,3′′-dimethyl-2,2′:5′,2′′-terthiophene]-5,5′′-diyl] (PMDPP3T) as the active layer blended with [6,6]-phenyl-C71-butyric acid methyl ester ([70] PCBM) with a various geometries of plasmonic silver nanostructures embedded on top of the back Ag layer of the OSCs to enhance the optical absorption for the longer wavelengths *i.e.* near NIR region. A well-known and extensively researched active layer material P3HT having a band gap of 1.9 eV restricts the optical absorption to wavelengths below 650 nm and is unable to exploit the NIR spectral regime of the sunlight. This results in only 22.4% of the total photons to be harvested from the solar spectrum. Previous studies have reported the maximum PCE is around 5% for OSCs having P3HT:PCBM as the AL polymer.^29,48,49^ In order to improve the PCE of OSCs, spectral coverage of the OSC needs to be extended near the NIR region by lowering the bandgap of active layer material. On the other hand, lowering the bandgap of polymer reduces the open-circuit-voltage (*V*_OC_) of OSC. Several studies have reported that the optimal bandgap for absorbing light in conjugated polymers is between 1.3 eV to 1.9 eV.^1,18,23–26,50^ Therefore, this research paper presents a new active layer polymer PMDPP3T which has an optimal bandgap of 1.3 eV, thereby showing a strong photoresponse up to 960 nm. The photoactive layer of PMDPP3T:PC70BM has not been previously used in a plasmonic enhanced single junction solar cell. However, Weiwei *et al.* presented a non-plasmonic single junction OSCs with a photoactive layer of PMDPP3T:PC70BM showing a maximum PCE of 7.0%.^50^

In this paper, we carry out FDTD numerical simulations to demonstrate the enhancement of the optical absorption and therefore the short circuit current density (*J*_SC_) of the OSCs containing PMDPP3T:PC70BM as the photo-active layer (AL) by embedding arrays of optimally sized plasmonic (Ag) nanostructures such as nano-spheres, nano-hemispheres, and nano-parabolas at the bottom of the solar cell such that these nanostructures are in contact with the back silver electrode. This leads to coupling of the incident light into SPPs as well as LSPs in these nanoparticles. This leads to enhanced scattering of light by optimally sized nanoparticles into the active layer, which leads to a strong optical absorption. Moreover, coupling of light into SPPs and LSPs leads to an enhancement of the electric field at the metal–dielectric interface inside the active layer. It has been demonstrated that the optical absorption of the proposed OSC having nano-parabolas at the back of the solar cell is significantly enhanced across wide range of spectral regime, which leads to a higher *J*_SC_ and PCE compared to a reference solar cell. The nano-parabola structure has not been previously employed in any kind of plasmonic solar cell – whether an organic solar cell or an inorganic solar cell. Moreover, this is the first time a nano-parabola structure has been employed for an OSC containing a low-bandgap polymer (PMDPP3T:PC70BM) as the photo-active layer.

## 2. Materials and methods

### 2.1. Organic solar cell materials and architecture

**Fig. 1:**
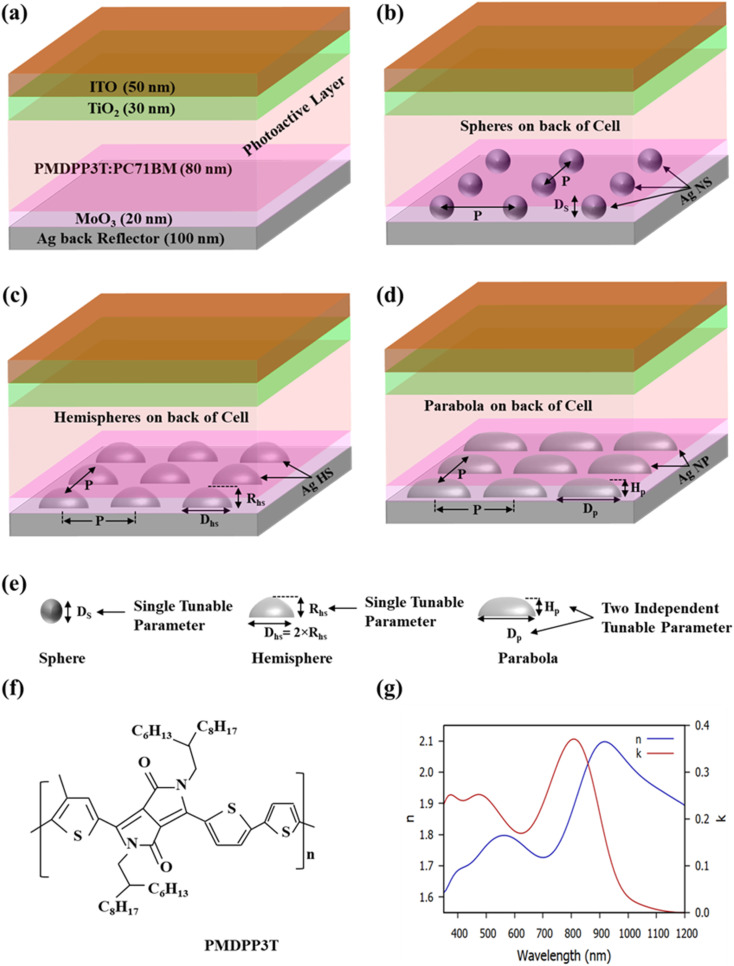
Schematic of the OSCs with and without plasmonic nanostructures: (a) Planar OSC *i.e.* OSC without plasmonic nanostructures (b) an OSC having a periodic Ag nano-spheres (Ag-NS) array on the top of the back Ag layer of the cell (c) an OSC with a periodic array of Ag nano-hemispheres (Ag-HS) on the top of the back Ag layer of the cell (d) an organic solar cell with a periodic array of Ag nano-parabola (Ag-NP) on the top of the back Ag layer of the cell (e) tunable parameters of plasmonic nanostructures (f) molecular structure of the new NIR-absorbing polymer (PMDPP3T) (g) real (*n*) and complex (*k*) refractive indices of the AL material PMDPP3T:PC70BM.

The device architecture of OSCs having plasmonic nanostructures at back of cell are shown in Fig. 1, wherein Fig. 1(a) show the reference OSC (without any plasmonic nanostructure) consisting of a 100 nm Ag layer as back reflector layer, a 20 nm MoO_3_ layer as anode buffer layer (ABL), a 80 nm PMDPP3T:PC70BM layer as photo-active layer (AL), 30 nm TiO_2_ layer as cathode buffer layer (CBL), and a 50 nm indium-tin-oxide (ITO) layer. Fig. 1(b), show the OSC with Ag nano-spheres (Ag-NS) on the top of the back Ag layer of OSC, an OSC with Ag nano-hemispheres (Ag-HS) on the top of the back Ag layer of OSC is shown in Fig. 1(c), an OSC having Ag-nano-parabola (Ag-NP) on the top of the back Ag layer of OSC is shown in Fig. 1(d). The tunable parameters of plasmonic nanostructures are shown in Fig. 1(e). The molecular structures of the new NIR absorbing polymer poly[[2,5-bis(2-hexyldecyl)-2,3,5,6-tetrahydro-3,6-dioxopyrrolo[3,4-*c*]pyrrole-1,4-diyl]-*alt*-[3′,3′′-dimethyl-2,2′:5′,2′′-terthiophene]-5,5′′-diyl] (PMDPP3T) and the real (*n*) and complex (*k*) refractive indices of the AL material PMDPP3T:PC70BM are presented in Fig. 1(f) and (g), respectively. However, the metallic nanoparticles embedded at the bottom of the OSC are extended inside the AL too. Recent studies have demonstrated that the spherical nanoparticles such as sphere, hemisphere, disc *etc.* shows a strong confined localized field in AL having thickness less than 100 nm.^51^ The optimally sized nanoparticles inside the active layer have demonstrated strong near field effect as well as enhanced scattering of light inside the active layer.^43,44^ Therefore, in the proposed OSCs the tunable parameters such as diameter of Ag nano-spheres (*D*_S_) and the periodicity *P* of Ag nano-spheres were optimized to maximize the *J*_SC_ and PCE of OSCs. For the plasmonic hemispheres array at the bottom of the cell, the tunable parameters, diameter of Ag-hemispheres (*D*_hs_) and the periodicity *P* of the Ag-HS array were optimized. For Ag nano-parabolas at the bottom of the cell, the tunable parameters, such as diameter of Ag nano-parabola (*D*_p_) and periodicity *P* of Ag nano-parabolas were optimized. However, in order to prevent from electrical shorting, the maximum height of nanoparticles placed at the bottom of the OSCs was fixed to 80 nm.

### 2.2. Numerical simulations of the organic solar cells

A commercial three-dimensional (3D) FDTD simulations software LUMERICAL FDTD solutions was used to calculate the plasmonic absorption enhancement, electromagnetic (EM) field distributions, and light scattering in the AL with optimally sized plasmonic nanoparticles embedded at the back of the solar cell. A TM polarized plane wave (polarization along *x*-directions) source in the spectral range from 350 nm to 1200 nm was normally incident on top surface of the OSCs from a distance 500 nm away from the top surface of OSCs structure. In order to simulate the proposed OSCs, the periodic boundary conditions were set in the *x* and *y* directions, and perfectly matched layer (PML) boundary conditions were set in *z*-directions (top and bottom boundaries). A non-uniform mesh size of 1 nm × 1 nm × 1 nm was taken for the simulation region of plasmonic nanostructure, and a mesh size of 3 nm × 3 nm × 3 nm was taken for the remaining region of OSCs. The Ag plasmonic nanoparticles were periodically placed at the back of the OSC with equal spacing in *x*–*y* directions. For FDTD simulations, the refractive indices (*n*, *k* values) of PMDPP3T:PC70BM were extracted from literature and plotted in Fig. 1(g).^50^

FDTD simulations were carried out to calculate the optical power absorbed in photoactive layer as a function of the incoming light wavelength in the range of 350 nm to 1200 nm. The time-averaged optical absorbed power in photoactive layer of OSCs was estimated by integrating the electric field.^52,53^5
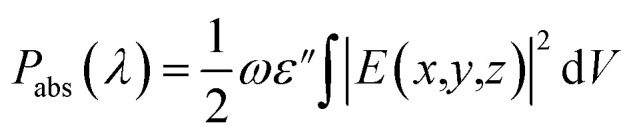
where *ε*′′ is the imaginary part of photoactive material dielectric constant, *ω* denotes the angular frequency of incident photon, *E* represents the electric field in the AL of OSC, and *V* is the volume of photoactive material. The volume integral (*V*) encompasses the entire photoactive medium of OSCs considering the fact that, – the power absorbed (*P*_abs_) in the photoactive layer of the OSCs is solely responsible for generation of electron–hole pair inside the active layer and contributes to the current generated in the OSCs. Thus, during the simulation, the index monitor was modelled to filter out the absorption of light by metallic nanoparticles. The optical absorption (*A*(*λ*)) in the photoactive layer is determined by normalizing the power absorbed by the AL of OSC to the incident input power:^52^6
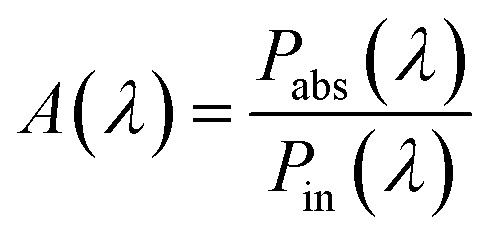
where *P*_abs_(*λ*) is the power absorbed by photoactive layer of OSC and *P*_in_(*λ*) is the incident input power. The *J*_SC_ is evaluated by multiplying the optical absorption *A*(*λ*) of the photoactive material with the solar irradiance spectrum AM1.5G and then integrated in entire wavelength range from 350 nm to 1200 nm. Considering that all the generated electron hole pairs in the AL contribute to the photocurrent, the *J*_SC_ of solar cell can be calculated as:7
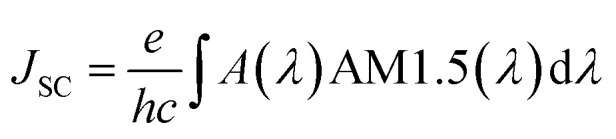
where *e* denotes the electronic charge, *h* denotes the Planck constant and *c* represents the speed of light in free space and AM1.5G is solar spectrum. The open circuit voltage (*V*_OC_) of a proposed OSCs made of a low band gap (band gap = 1.3 eV) photoactive material (PMDPP3T:PC70BM) having donor and acceptor blend is^54^8
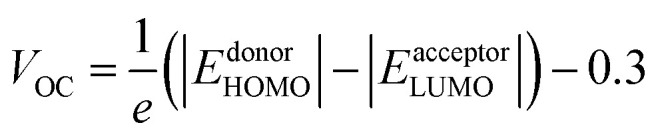
where, *e* is the electronic charge. An empirical value of 0.3 V is added in the ref. 53. For the active layer of proposed OSCs *E*^PMDPP3T^_HOMO_ = 5.2 eV, and 
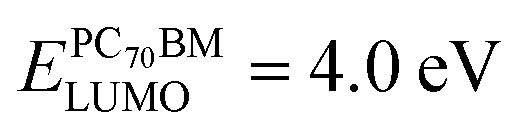
.^50,55^ Thus, the *V*_OC_ = 0.9 V.

Finally, PCE of proposed OSCs was evaluated by using the formula,9
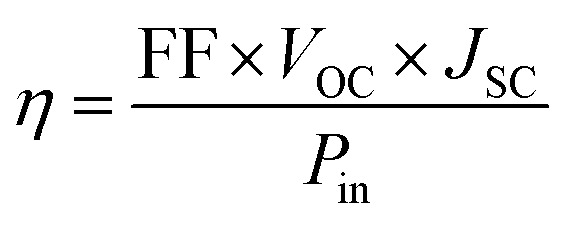
where, solar cell fill factor is FF, and *P*_in_ is the incident power (100 mW cm^−2^).

## 3. Results and discussion

This section demonstrated the plasmonic enhancement of photovoltaic characteristics of proposed OSCs in terms of *J*_SC_ and PCE due to the addition of the Ag-NS arrays, Ag-HS arrays, and Ag-NP arrays at the bottom of the OSCs. Using the LUMERICAL FDTD simulation software, the solar cell performances *J*_SC_ and PCE were analyzed for the four different device structures shown in Fig. 1(a)–(d). The *J*_SC_ and PCE of the OSCs were maximized by using low band gap active layer material and by varying the tunable parameters such as diameter and height of plasmonic nanoparticles embedded at the back of the cell. However, to prevent from the electrical shorting the maximum height of nanoparticles present at the bottom of the cell was kept constant to 80 nm.

### 3.1. Optimization of bottom layer nanoparticle parameters of the OSCs

In order to increase the light absorption in AL of OSCs, the spherical nanoparticles have been placed into the AL. The spherical nanoparticles have been gaining great attention due to their simple and low-cost fabrication process. Colloidal metal NPs having a spherical shape can be incorporated in polymer based OSCs by mixing them in polymeric solutions and employing spin coating. Moreover, nanostructures of plasmonic metals such as gold and silver can conveniently be fabricated using nano-sphere lithography or nanoimprint lithography.^56,57^

**Fig. 2:**
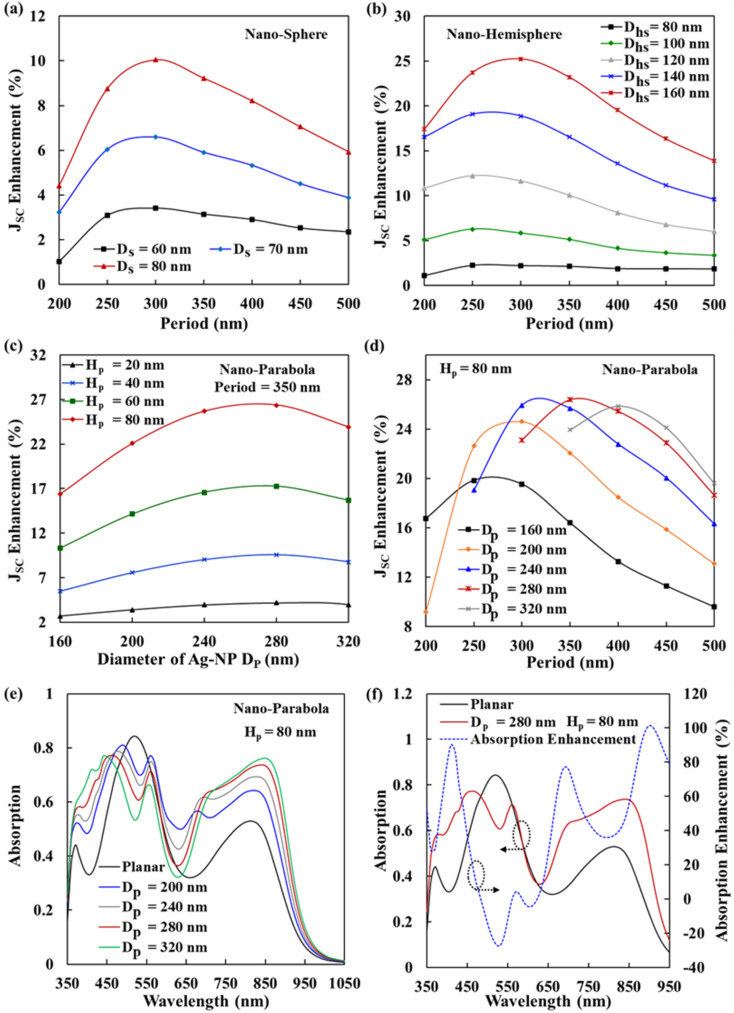
(a)–(d) FDTD simulation results demonstrating the effects of varying the structural parameters and the periodicity of plasmonic NPs on the values of the enhancements in the *J*_SC_ (compared to the *J*_SC_ of planar OSC) of the OSCs containing these plasmonic NPs on the top of the back Ag layer of the OSC. The periodicity (*P*) of the NPs must be greater than the diameter of the NP. Therefore, the curves in plot (d) have different starting points. Plot (e) shows the absorption spectra as a function of the nano-parabola diameter (*D*_p_). Plot (f) show the optimized structure absorption (left *y*-axis) and absorption enhancement (right *y*-axis) as a function of wavelength for a solar cell with 2D array of nano-parabola at the back of the solar cell.

The two fundamental mechanisms – light scattering and near-field enhancement – are responsible for absorption enhancement in the active material of OSCs having spherical nanoparticles embedded into the AL. The dominance of each mechanism depends on particle size and shape. Therefore, the FDTD method is used to optimize the structural parameters of the bottom NPs to enhance the *J*_SC_ and PCE of the proposed OSCs. Fig. 2 illustrates the optimization of the structural parameters of the different plasmonic NP arrays embedded at the back of the OSCs. Fig. 2(a) shows the improvement in *J*_SC_ of OSCs due to the Ag-NS arrays for different diameters (*D*_S_) as a function of Ag-NS period. It can clearly be observed from Fig. 2(a) that the maximum value of *J*_SC_ enhancement of 10.09% was achieved for an Ag-NS period (*P*) of 300 nm and a Ag-NS diameter (*D*_S_) of 80 nm. It has to be mentioned that the *J*_SC_ of solar cell increases with the diameter of nano-spheres irrespective of the period. However, in order to prevent electrical shorting of the device, the diameter of nano-spheres embedded at the back of the solar cell should not be increased beyond the length of ABL + AL. Therefore, the maximum diameter of nano-spheres embedded at the back of the cell has to be restricted to 80 nm (less than the length of ABL + AL). Although nano-spheres of such small dimensions (and covering only 2.9% of the volume of ABL + AL) can enhance the near-field around the nano-spheres, but the light scattering by the plasmonic nanoparticles is underachieved. Hence, the maximum possible absorption enhancement in active layer is not achieved.

In order to enhance the light scattering phenomena in AL of OSC same class of spherical nanoparticles *i.e.* hemispheres have been introduced at the bottom of the OSC which are able to cover more volume of thin AL compared to nano-spheres. Fig. 2(b) shows the enhancement in *J*_SC_ of OSC due to the Ag-nano-hemispheres (Ag-HSs) array for different diameter (*D*_hs_) as a function of Ag-HSs period. We can clearly observe from Fig. 2(b) that the maximum value of *J*_SC_ enhancement 25.22% was achieved for a nano-hemisphere period (*P*) of 300 nm and a nano-hemisphere diameter (*D*_hs_) of 160 nm. It has to be noted that the *J*_SC_ enhancement of solar cell having Ag hemispheres (*D*_hs_ = 160 nm) at the bottom of the OSC is much higher than the *J*_SC_ enhancement of solar cell having Ag spheres at the bottom of the OSC. Fig. 2(b) clearly shows that the *J*_SC_ enhancement increases with the diameter of hemispheres. The structural limitations associated with hemisphere that the diameter and height both are related to each other and has to be tuned with single parameter (height of hemisphere is half of diameter). Hence, the maximum diameter of hemisphere has to be restricted to 160 nm (maximum height is fixed to 80 nm) which occupied 11.91% of volume of ABL + AL. Therefore, the *J*_SC_ of solar cells can be further maximize by introducing the same class of nanostructures which have two independent tunable parameters in *x*–*z* direction.

This paper introduces a parabola nanostructure at the bottom of the OSC for the first time to obtain the extremely large light absorption enhancement in the AL. Although the parabola nanostructure has a similar geometrical construction to that of a hemisphere, it has two independent tunable parameters – diameter and the height. Fig. 2(d) shows the *J*_SC_ enhancement of OSC due to the parabola nanostructures array for different diameters (varying from 160 nm to 320 nm) as a function of the nano-parabola period. It is seen from Fig. 2(d) that the highest value of *J*_SC_ enhancement of 26.41% was achieved for a nano-parabola period of 350 nm and a nano-parabola diameter of 280 nm. However, the height of nano-parabola is kept constant 80 nm. Considering the optimal period of nano-parabola 350 nm, Fig. 2(c) shows the enhancement in *J*_SC_ of OSC having nano-parabola at the bottom of the OSC as a function of bottom nano-parabola diameter. We can observe from Fig. 2(c) that the *J*_SC_ enhancement increases with nano-parabola diameter varied from 160 nm to 280 nm and then decreases. Fig. 2(e) shows the absorption in photoactive layer as a function of wavelength for an OSC having nano-parabola on top of the back Ag layer of the cell for different values of the nano-parabola diameter (*D*_p_). Fig. 2(e) also demonstrates that the OSC structure having the nano-parabolas at the back of the cell has higher absorption compared to a planer OSC for wavelengths ranging from 350 nm to 480 nm and for wavelengths longer than 650 nm. Fig. 2(f) presents the absorption enhancement spectra as a function of wavelength for optimized structure OSC having nano-parabolas on the back layer of the OSC. The strong absorption enhancement is observed in photoactive layer of OSCs due to enhanced scattering of light by optimally sized metal nanoparticles inside the AL. Thus, it is demonstrated that the OSC having nano-parabolas at the back of the cell shows highest enhancement in the absorption and *J*_SC_ compared to other OSC structures.

### 3.2. Comparative analysis of a planar OSC, an OSC having Ag-NS on the top of the back Ag layer, an OSC having Ag-HS on the top of the back Ag layer, and an OSC containing Ag-nano parabola on the top of the back Ag layer

**Fig. 3:**
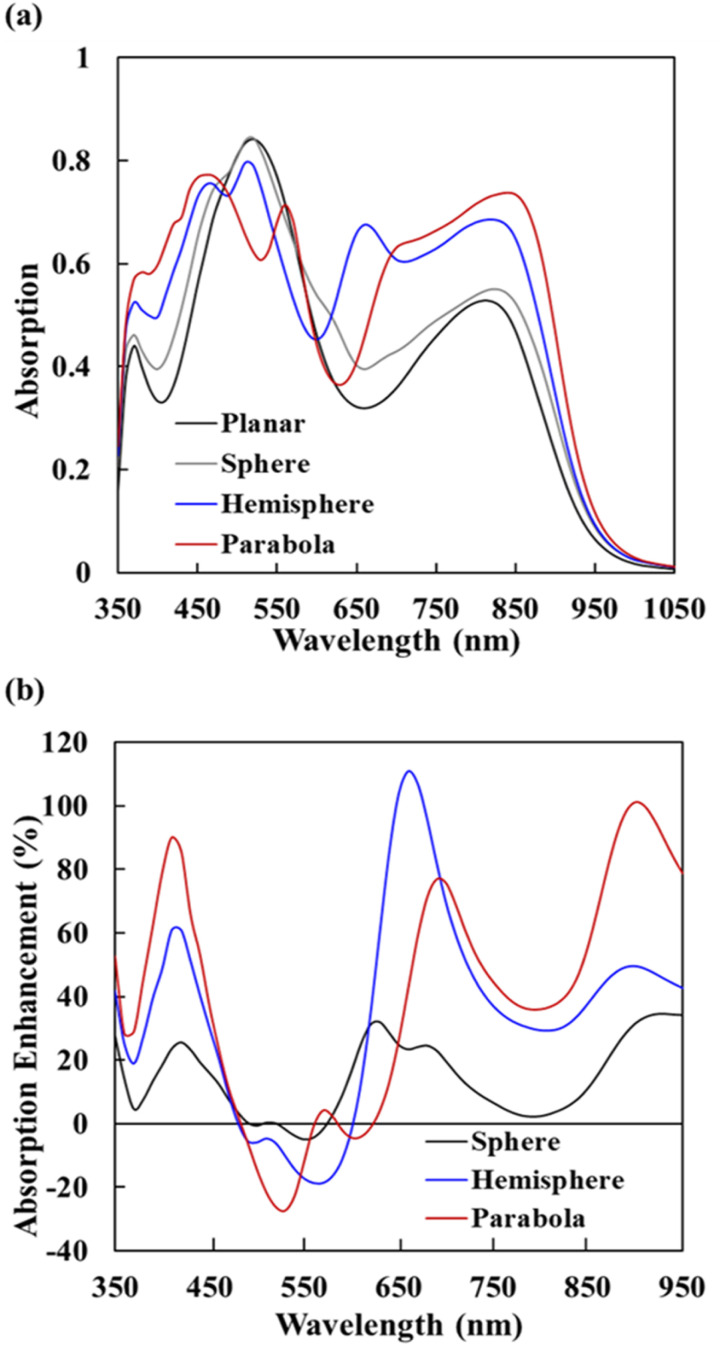
(a) Absorption spectra of the OSCs containing – 2D array of Ag NSs on the top of the back Ag layer of OSC (silver line), 2D array of Ag-HSs on the top of the back Ag layer of OSC (blue line), and 2D array of Ag nano parabolas on the top of the back Ag layer of OSC (red line) and a planar OSC (black line), (b) absorption enhancement spectra of OSCs containing – 2D array of Ag nano spheres (black line), 2D array of Ag nano hemispheres (blue line), and 2D array of Ag nano parabola (red line) at the back of the OSC (the enhancement is calculated with respect to planar OSC).

The absorption spectra of OSCs containing different optimally sized plasmonic nanostructures are plotted as a function of wavelength and shown in Fig. 3(a). The absorption spectrum of a planar OSC (an OSC having no plasmonic nanostructure) is also included in Fig. 3(a) for comparison. Fig. 3(b) shows the absorption enhancement spectra of OSCs having different optimally sized plasmonic nanostructures array at the back of the cell. The results demonstrate that the inclusion of the plasmonic nanostructures array increases the light absorption in the AL over a wide spectral region. Fig. 3(a) and (b) demonstrate an absorption enhancement for wavelengths between 350 nm and 490 nm (with 25.70% absorption enhancement at 420 nm) for the OSCs having nano-spheres at the back of the solar cell, an absorption enhancement for wavelength between 350 nm and 480 nm (with 60.90% absorption enhancement at 410 nm) for the OSCs having nano-hemispheres at the back of the cell, and an absorption enhancement for wavelength between 350 nm and 480 nm (with 90.21% absorption enhancement at 410 nm) for the OSCs having nano-parabolas at the back of the cell. Another strong absorption enhancement is registered for longer wavelengths (*i.e.*, for wavelengths greater than 600 nm) for OSCs with nano-hemispheres and nano-parabolas-with a major peak of 110.94% enhancement at 660 nm and a minor peak of 49.57% enhancement at 900 nm for the OSCs having 2D nano-hemispheres arrays at the back of the cell, with a major peak of 101.28% enhancement at 900 nm and a minor peak of 77.03% enhancement at 690 nm for the OSCs having 2D nano-parabolas array at the back of the cell. These enhancement mechanisms in photoactive layer due to the incorporation of metallic nanoparticles array such as nano spheres, nano hemispheres, and nano parabolas inside the AL of OSCs exploits the near-field enhancement (strongly confined field of the localized surface plasmon resonance) and efficient far-field light scattering inside the AL.

**Fig. 4:**
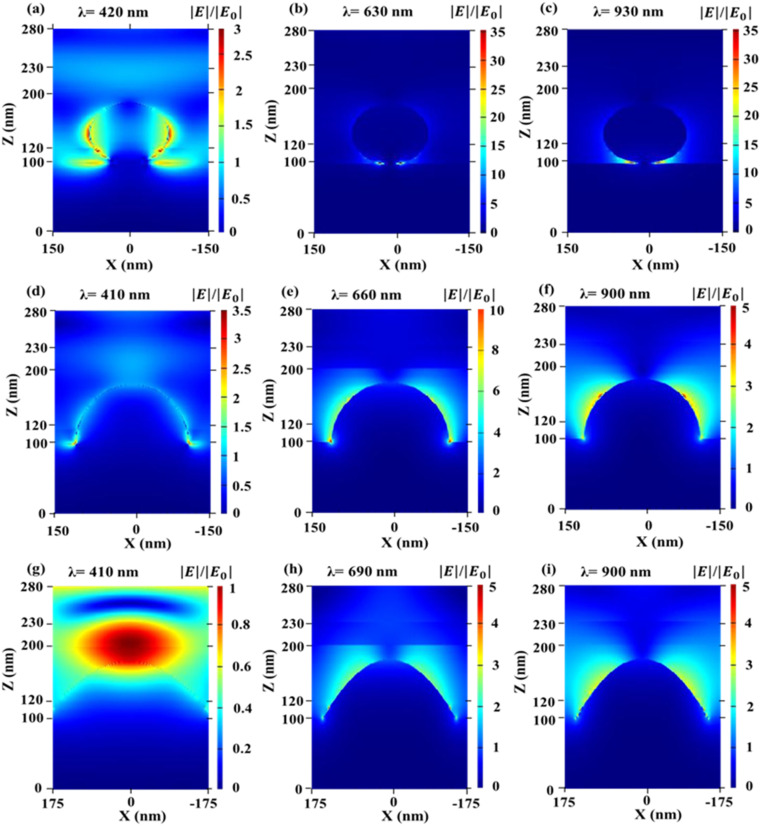
(a)–(c) Normalized electric field distributions in the *x*–*z* plane at a wavelength of 420 nm, 630 nm, and 930 nm for an OSC having 2D array of Ag nano-spheres at the back of the cell. (d)–(f) Normalized electric field distributions in the *x*–*z* plane at a wavelength of 410 nm, 660 nm, and 900 nm for an OSC having 2D array of Ag nano-hemispheres at the back of the cell. (g)–(i) Normalized electric field distributions in the *x*–*z* plane at a wavelength of 410 nm, 690 nm and 900 nm for an OSC having a 2D array of Ag nano parabolas on the top of the back Ag layer of the cell. These graphs were plotted using the optimized values of diameters and periods. For the OSC having a 2D array of Ag nano-spheres on the top of the back Ag layer of the cell, the optimized values of sphere diameter (*D*_S_) and periodicity *P* were used to be 80 nm and 300 nm, respectively. For the OSC having a 2D array of Ag nano-hemispheres on the top of the back Ag layer of the cell, the optimized values of diameter (*D*_hs_) and periodicity *P* were used to be 160 nm and 300 nm, respectively. For the OSC having a 2D array of Ag nano parabolas on the top of the back Ag layer of the cell, the optimized values of diameter (*D*_p_), height (*h*), and periodicity *P* were used to be 280 nm, 80 nm, and 350 nm, respectively.

**Fig. 5:**
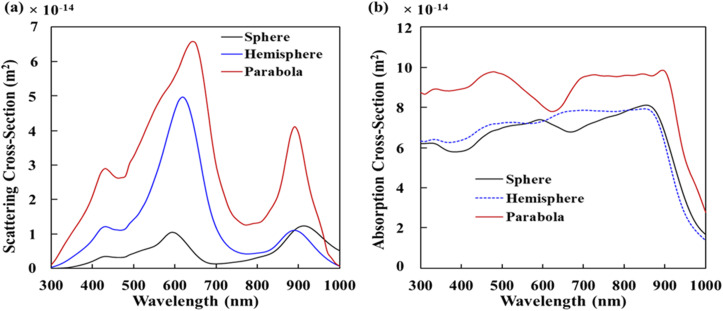
(a) Scattering cross section and (b) absorption cross section of for different optimized plasmonic NPs. The NPs were embedded at the back of the solar cell and extends inside the active layer too. The diameter of plasmonic spheres, hemispheres, and parabolas were taken to be 80 nm, 160 nm, and 280 nm, respectively. The periodicity for Ag spheres and Ag hemispheres were taken to be 300 nm, while the periodicity for Ag parabolas was taken to be 350 nm.

Fig. 4(a)–(i) shows the normalized *E*-field (|*E*|/|*E*_0_|) distribution of OSCs having the 2D periodic array of Ag NPs (nano spheres, nano hemispheres, and nano parabolas) at the back of the cells in *x*–*z* plane. Fig. 4(a)–(c) shows the normalized *E*-field distribution of OSCs having the 2D periodic array of Ag-NS at the back of the cell in *x*–*z* plane, Fig. 4(d)–(f) shows the normalized *E*-field distribution of OSCs having the 2D periodic array of Ag-HSs at the back of the cell, and Fig. 4(g)–(i) shows the normalized *E*-field distribution of OSCs containing the 2D periodic array of nano-parabolas at the back of the cell. The normalized field distribution in the vicinity of nano sphere, nano hemisphere, and nano parabola shows several LSPR modes such as dipole, quadrupole, and octupole at different wavelengths. It can been observed from Fig. 4 that the near-field enhancement effect (due to localized surface plasmons) is more prevalent for nano spheres (nanoparticles having a small diameter, *i.e.* for *D*_S_ = 80 nm). On the other hand, hemispheres and nano parabolas which have a higher diameter (*D*_hs_ = 160 nm and *D*_pc_ = 280 nm). The scattering and absorption cross section of spherical nanoparticles are calculated by eqn (1) and (2). As the diameter of the NPs (hemispheres and parabolas) increases, the light scattering increases significantly more than the absorption. Therefore, the enhancement due to light scattering is more prevalent for the OSCs containing nano hemispheres and nano parabolas (large sized nanoparticles) at the back Ag layer of the cell. On the other hand, the near-field enhancement effect is prevalent for the OSCs having spheres (small size nanoparticles) at the back Ag layer of the cell. As the optimized diameters of nano hemispheres and nano parabolas are much higher than the nano-spheres, the scattering effect will become stronger for nano hemispheres and nano parabolas according to Mie theory. Higher diameter nano hemispheres and nano parabola structures have large scattering cross sections, and a large amount of light is scattered into the photoactive layer. Moreover, the enhanced scattering of light inside the AL of the OSC makes the effective optical path length substantially longer than the actual thickness of the AL. This leads to an increased absorption of light in the AL. Hence, Fig. 3(a) and (b) demonstrate that the light absorption enhancement is higher for OSCs having 2D array of nano hemispheres and nano parabolas on top of the back Ag layer of the cell compared to the OSCs having 2D array of nano spheres on top of the back Ag layer of the cell. The light scattering and absorption cross sections for optimized plasmonic NPs such as sphere, hemisphere, and parabola are shown in Fig. 5(a) and (b), respectively. It has clearly observed from Fig. 5(a) and (b) that the scattering cross section as well as the absorption cross section is the highest for the nano parabola over a wide wavelength range due to the excitation of LSPRs at several wavelengths in the case of a nano parabola. The enhancement of the scattering cross section implies that the parabola nanostructure scatters more light back into the AL of OSC, leading to an enhanced absorption of light in the AL of the OSC.

Therefore, the nano-parabola array leads to a higher enhancement in the *J*_SC_ of the OSCs, which was shown in Fig. 2. Fig. 3(b) shows three enhanced peaks in the absorption spectrum at 410 nm, 690 nm, and 900 nm for the OSCs having nano parabolas on top of the back Ag layer of the cell which are in good correlation with the scattering cross section peaks of nano-parabola structure. On the other hand, we can observe from Fig. 5(b) that the peaks of the absorption cross-section of the of parabola nanostructure are not in good correlation with the peaks in the absorption spectrum of the OSC. This implies that scattering of light by the parabola nanostructures has a significantly greater impact in enhancing the light absorption in the AL of OSC as compared to the near-field enhancement by the NPs. It can also be observed from Fig. 5(a) that the nano hemispheres have a higher scattering cross section as compared to the nano spheres. Therefore, the OSCs with plasmonic hemispheres shows higher *J*_SC_ compared to the OSCs with plasmonic spheres.

### 3.3. Power conversion efficiency of the various structure OSCs

**Fig. 6:**
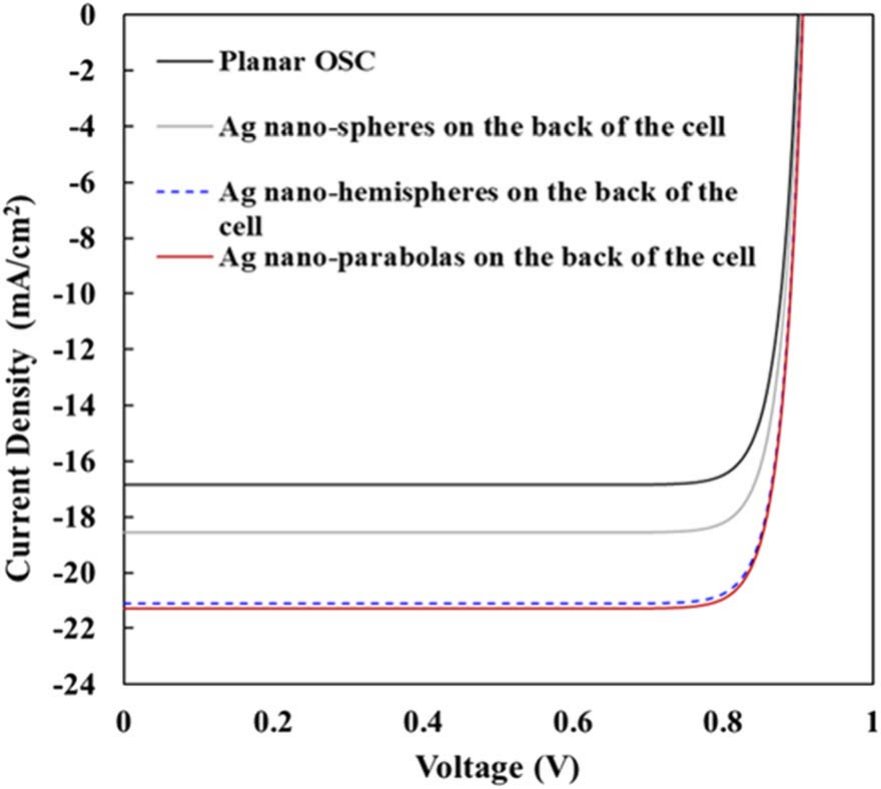
*J*–*V* characteristics of planar OSC (black line), OSC having Ag-NS on top of the back Ag layer of the cell (silver line), OSC having Ag-HS on top of the back Ag layer of the cell (blue line), and OSC having Ag-NP on top of the back Ag layer of the cell (red line). The optimized nanostructure parameters (optimized value of diameters and periods) have been used for the calculation of *J*_SC_ of various structure solar cells. For the array of plasmonic nano-spheres, the values of sphere diameter (*D*_S_) and periodicity *P* were taken to be 80 nm and 300 nm, respectively. For the array of plasmonic nano-hemispheres, the values of diameter (*D*_hs_) and periodicity *P* were taken to be 160 nm and 300 nm, respectively. For the array of plasmonic nano-parabolas, the values of diameter (*D*_p_), height (*h*), and periodicity *P* were taken to be 280 nm, 80 nm, and 350 nm, respectively.

This section analyses the *J*–*V* characteristics of the different OSCs shown in Fig. 1. The *J*–*V* characteristics of different OSCs were analyzed taking the operating conditions to be ideal. Under ideal operating conditions, the series resistance and shunt resistance are considered to be zero and infinite, respectively, and the *J*–*V* characteristics of different structure OSCs were assessed using the ideal diode equation.^58,59^10
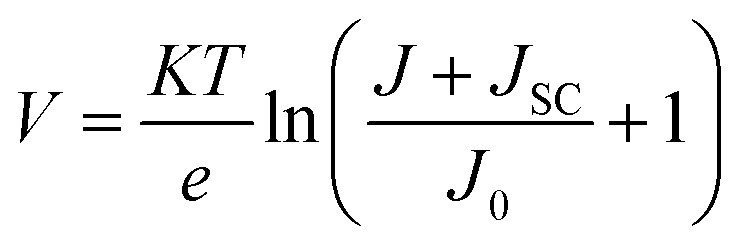
where *e* denotes the electronic charge, *k* denotes the Boltzmann constant, *T* = 300 K represents the ambient temperature, and *J*_0_ represents the saturation dark current density. The open circuit voltage (*V*_OC_) for the BHJ photoactive layer (PMDPP3T:PC70BM) used in this paper was estimated from eqn (8), *i.e.*, *V*_OC_ = 0.9 V and the *J*_SC_ of planar OSC was evaluated from eqn (7), *i.e.*, *J*_SC_ = 16.85 mA cm^−2^. When the OSCs operates under open circuit condition *i.e.*, *V* = *V*_OC_ and *J* = 0, the dark saturation current density (*J*_0_) = 1.29 × 10–13 A m^−2^ was evaluated for planar OSC and it was used as a constant for plotting the *J*–*V* curves of the different structure OSCs depicted in Fig. 6. Finally, using the realistic value of fill factor (FF) of proposed OSCs = 0.66,^50^ the PCE of organic solar cells were evaluated using eqn (9), where the input power (*P*_in_) = 100 mW cm^−2^ and open circuit voltage (*V*_OC_) = 0.9 V of proposed OSCs, the eqn (9) minimizes to:

11 *η* = 0.009 × FF × *J*_SC_

The photovoltaic characteristics of various structure OSCs shown in Fig. 1 were evaluated using the realistic value of FF and the numerical values are presented in Table 1. It was observed that for the OSCs having a 2D periodic array of Ag nano-spheres on top of the back Ag layer of the cell (for optimized geometries of the nano-spheres), there is a *J*_SC_ and PCE enhancement of 10.09% and 10.09%, respectively, compared to that of a planar OSC. It was also observed that for an OSC having a 2D periodic array of Ag nano-hemispheres on top of the back Ag layer of the cell (for optimized geometries of the nano hemispheres), there is a *J*_SC_ and PCE enhancement by 25.22% and 25.17%, respectively, compared to that of a planar OSC. Finally, it was observed that for an OSC having 2D periodic array of parabola nanostructures (for optimized geometries of the parabola nanostructures) on top of the back Ag layer of the cell, there is a significant enhancement of the *J*_SC_ and the PCE by 26.41% and 26.37%, respectively, compared to that of a planar OSC.

**Table 1:** Photovoltaic characteristics of the OSCs containing plasmonic nano-spheres, nano-hemisphere, and nano-parabola on top of the back Ag layer of the cell

	*J*_SC_ (mA cm^−2^)	*J*_SC_ enhancement (%)	*V*_OC_ (V)	Fill factor (%)	PCE (*η*) (%)	Relative PCE enhancement (%)
Planar OSC	16.85	NA	0.9	66	10.01	NA
Ag nano spheres on top of the back Ag layer of the OSC	18.55	10.09	0.9	66	11.02	10.09
Ag nano hemispheres on top of the back Ag layer of the OSC	21.10	25.22	0.9	66	12.53	25.17
Ag nano parabola on top of the back Ag layer of the OSC	21.30	26.41	0.9	66	12.65	26.37

The PCE is primarily dependent on the *V*_OC_, *J*_SC_, and FF of solar cell. Table 1 presents the PCE of different OSCs which was calculated using the same realistic fill factor = 0.66. The introduction of plasmonic nanoparticles at the back of the OSC may further enhance the FF of OSCs.^60,61^ Thus, the proposed plasmonic OSCs may offer higher PCE compared to the PCE estimated in Table 1.

The OSCs proposed and simulated in this paper can easily be fabricated using a low-cost and large-area fabrication process. In order to fabricate the plasmonic nanostructures such as the arrays of silver nano-parabola being proposed in this paper, a combination of several processes such as nano-sphere lithography, reactive ion etching, and argon ion milling may need to be employed.^62^ If the plasmonic nanostructures are developed on the silicon substrates, the substrate wafers can first be coated with a thin film of silver (∼200 nm), with a thin adhesion layer (5 nm) of titanium deposited between the silicon substrate and the silver film. This can be followed by self-assembly of polystyrene nano-spheres (diameter ∼500 nm) on the silver film, wherein the polystyrene nano-spheres act as an etch mask. This can be followed by reactive ion etching with an oxygen plasma to get a reduction in the size of the nano-spheres. This can be followed by argon ion milling first of the polystyrene spheres themselves and then the underlying silver film to form nano-parabola structures, with a polystyrene cone on top of the nano-parabola structures of silver. This can finally be followed by reactive ion etching with an oxygen plasma to remove the polystyrene cone on top of the nano-parabola structures of silver and thereby obtain the desired array of silver nano-parabolas. In order to form the OSC containing the nano-parabola shaped plasmonic nanostructures, deposition (using sputter deposition or thermal evaporation) of a thin MoO_3_ layer (20 nm) can be carried out. The polymeric materials forming the AL (PMDPP3T:PC70BM) of the OSC can then either be spin-coated, sprayed, or dip-coated on top of the MoO_3_ coated plasmonic nanostructures. This can be followed by deposition (using sputter deposition or thermal evaporation) of a thin TiO_2_ layer (30 nm). Finally, this can be followed by sputter deposition of the top ITO layer (50 nm).

## 4. Conclusion

This paper employs FDTD simulations to describe the plasmonic enhancement of the *J*_SC_ and the PCE for a single junction solar cell that employs a relatively new low band gap photoactive polymer (PMDPP3T) that is capable of absorbing the incident light up to a wavelength of 960 nm. The introduction of plasmonic nanostructures at the back of the OSC significantly enhances the overall photovoltaic characteristics of the OSC. This paper discusses three different nanostructured OSCs–OSCs containing plasmonic nano-spheres on top of the back Ag layer of the cells, OSCs containing plasmonic nano-hemispheres on top of the back Ag layer of the cells, and OSCs containing plasmonic nano-parabolas on top of the back Ag layer of the cells. It was demonstrated that the OSCs having nano-parabolas on top of the back Ag layer of the cells show a significant absorption enhancement in photoactive layer in the wide range of spectral regime due to the higher scattering of light inside the AL, resulting in a significant enhancement in *J*_SC_ and PCE. An optimized OSC having 2D array of Ag nano-parabolas on top of the back Ag layer of the cell showed a 26.41% enhancement in the *J*_SC_ as compared to the *J*_SC_ of the planar OSC (OSC having without plasmonic nanostructure), whereas optimized OSCs having 2D array of Ag nano-hemispheres, and 2D array of Ag nano-spheres on top of the back Ag layer of the cells showed *J*_SC_ enhancements of 25.22%, and 10.09%, respectively, as compared to that of a planar OSC. It was demonstrated that the enhancement in the absorption of OSCs can be mainly attributed due to the light scattering by metal nanostructures, which is the main mechanism behind enhanced light trapping inside the OSCs.

## Appendix A

### Optimization of diameter and height of nano-parabolas present at the back of the OSC

**Fig. 7:**
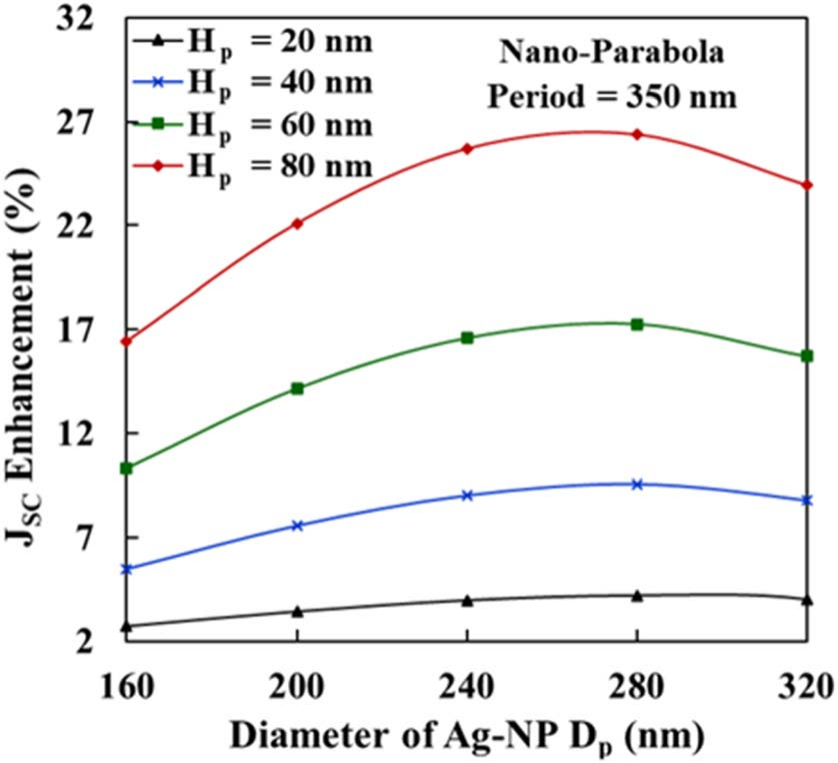
*J*_SC_ enhancement in the OSC by employing the 2D array of nano-parabolas on the top of back Ag layer as a function of back parabola diameter (*D*_p_) for varying height − *H*_p_ = 20 nm, 40 nm, 60 nm, and 80 nm.

To obtain the maximum enhancement in *J*_SC_ due to the presence of a bottom parabola array (parabola array present at the top of the back Ag layer), the diameter and height of the bottom parabolas were varied from 160 nm to 320 nm and from 20 nm to 80 nm, respectively. Fig. 7 presents the *J*_SC_ enhancement as a function of bottom parabola diameter *D*_p_, when the bottom parabola cone height (*H*_p_) was varied from 20 nm to 80 nm. A maximum *J*_SC_ enhancement of 26.41% was obtained for *D*_p_ = 280 nm and *H*_p_ = 80 nm. The *J*_SC_ of solar cell increases with the height of parabola cone (*H*_p_) irrespective of the parabola cone diameter (*D*_p_). However, to prevent from electrical shorting of the device, maximum height of parabola embedded on the top of back Ag layer of the cell must be less than ABL + AL length. Therefore, the maximum height of parabola embedded at the back of the cell is restricted to 80 nm. Further, to optimize the solar cell (solar cell having nano-parabola on the top of back Ag layer), the height of parabola is kept constant 80 nm and the diameter of the parabola is varied from 160 nm to 320 nm. We can observe from Fig. 7 that the *J*_SC_ enhancement increases when the parabola diameter is varied from 160 nm to 280 nm and then decreases for higher values of the parabola diameter.

## Conflicts of interest

There are no conflicts to declare.

## Supplementary Material

## References

[cit1] Atwater H. A., Polman A. (2010). Plasmonics for improved photovoltaic devices. Nat. Mater..

[cit2] Chen J.-T., Hsu C.-S. (2011). Conjugated polymer nanostructures for organic solar cell applications. Polym. Chem..

[cit3] Bhat P. K., Das S. R., Pandya D. K., Chopra K. L. (1979). Back illuminated high efficiency thin film Cu_2_S/CdS solar cells. Sol. Energy Mater..

[cit4] Shin B., Gunawan O., Zhu Y., Bojarczuk N. A., Chey S. J., Guha S. (2013). Thin film solar cell with 8.4% power conversion efficiency using an earth abundant Cu_2_ZnSnS_4_ absorber. Prog. Photovolt.: Res. Appl..

[cit5] Guo, Zhu Y., Gunawan O., Gokmen T., Deline V. R., Ahmed S. (2014). Electrodeposited Cu_2_ZnSnSe_4_ thin film solar cell with 7% power conversion efficiency. Prog. Photovolt.: Res. Appl..

[cit6] O’Regan B., Gratzel M. (1991). A low-cost, high-efficiency solar cell based on dye-sensitized colloidal TiO_2_ films. Nature.

[cit7] Ye M., Zheng D., Lv M., Chen C., Lin C., Lin Z. (2013). Hierarchically structured nanotubes for highly efficient dye-sensitized solar cells. Adv. Mater..

[cit8] Feldt S. M., Gibson E. A., Gabrielsson E., Sun L., Boschloo G., Hagfeldt A. (2010). Design of organic dyes and cobalt polypyridine redox mediators for high-efficiency dye-sensitized solar cells. J. Am. Chem. Soc..

[cit9] Barnham K. W. J., Duggan G. (1990). A new approach to high-efficiency multi-band gap solar cells. J. Appl. Phys..

[cit10] Pan Z., Mora-Sero I., Shen Q., Zhang H., Li Y., Zhao K. (2014). *et al.*, High-efficiency green quantum dot solar cells. J. Am. Chem. Soc..

[cit11] Chuang C.-H. M., Brown P. R., Bulovic V., Bawendi M. G. (2014). Improved performance and stability in quantum dot solar cells through band alignment engineering. Nat. Mater..

[cit12] Tang C. W. (1986). Two-layer organic photovoltaic cell. Appl. Phys. Lett..

[cit13] He Z., Zhong C., Su S., Xu M., Wu H., Cao Y. (2012). Enhanced power-conversion efficiency in polymer solar cells using an inverted device structure. Nat. Photonics.

[cit14] Zhou H., Yang L., You W. (2012). Rational design of high-performance conjugated polymers for organic solar cells. Macromolecules.

[cit15] Kojima A., Teshima K., Shirai Y., Miyasaka T. (2009). Organometal halide perovskites as visible-light sensitizers for photovoltaic cells. J. Am. Chem. Soc..

[cit16] Lee M. M., Teuscher J., Miyasaka T., Murakami T. N., Snaith H. J. (2009). Efficient hybrid solar cells based on meso-superstructured organometal halide perovskites. Science.

[cit17] Teridi M. A. M., Sookhakian M., Basirun W. J., Zakaria R., Schneider F. K., da Silva W. J. (2015). *et al.*, Plasmon enhanced organic devices utilizing highly ordered nanoimprint gold nanodisks and nitrogen doped graphene. Nanoscale.

[cit18] Green M. A., Emery K., Hishikawa Y., Warta W., Dunlop E. D. (2016). Solar cell efficiency tables (version 47). Prog. Photovolt. Res. Appl..

[cit19] Saliba M., Orlandi S., Matsui T., Aghazada S., Cavazzini M., Correa-Baena J.-P. (2016). *et al.*, A molecularly engineered hole-transporting material for efficient perovskite solar cells. Nat. Energy.

[cit20] Guerrero A., You J., Aranda C., Kang Y. S., Garcia-Belmonte G., Zhou H. (2016). *et al.*, Interfacial degradation of planar lead halide perovskite solar cells. ACS Nano.

[cit21] Berhe T. A., Su W.-N., Chen C.-H., Pan C.-J., Cheng J.-H., Chen H.-M. (2016). *et al.*, Organometal halide perovskite solar cells: degradation and stability. Energy Environ. Sci..

[cit22] Han Y., Meyer S., Dkhissi Y., Weber K., Pringle J. M., Bach U. (2015). *et al.*, Degradation observations of encapsulated planar CH3NH3PbI3 perovskite solar cells at high temperatures and humidity. J. Mater. Chem. A.

[cit23] Hoppe H., Sariciftci N. S. (2004). Organic solar cells: An overview. J. Mater. Res..

[cit24] Hauch J. A., Schilinsky P., Choulis S. A., Childers R., Biele M., Brabec C. J. (2008). Flexible organic P3HT: PCBM bulk-heterojunction modules with more than 1 year outdoor lifetime. Sol. Energy Mater. Sol. Cells.

[cit25] Peters C. H., Sachs-Quintana I. T., Kastrop J. P., Beaupre S., Leclerc M., McGehee M. D. (2011). High efficiency polymer solar cells with long operating life-times. Adv. Energy Mater..

[cit26] Gan Q., Bartoli F. J., Kafafi Z. H. (2013). Plasmonic-enhanced organic photovoltaics: Breaking the 10% efficiency barrier” Adv. Mater.

[cit27] Yang X., Loos J., Veenstra S., Verhees W., Wienk M., Kroon J., Michels M., Janssen R. (2005). Nanoscale morphology of high-performance polymer solar cells. Nano Lett..

[cit28] Peumans P., Yakimov A., Forrest S. R. (2003). Small molecular weight organic thin-film photodetectors and solar cells. J. Appl. Phys..

[cit29] Li G., Shrotriya V., Huang J., Yao Y., Moriarty T., Emery K., Yang Y. (2005). High-efficiency solution processable polymer photovoltaic cells by self-organization of polymer blends. Nat. Mater..

[cit30] Lu L., Yu L. (2014). Understanding low bandgap polymer PTB7 and optimizing polymer solar cells based on IT. Adv. Mater..

[cit31] De Chen J., Cui C., Li Y. Q., Zhou L., Ou Q. D., Li C., Li Y., Tang J. X. (2015). Single-junction polymer solar cells exceeding 10% power conversion efficiency. Adv. Mater..

[cit32] Mayer A. C., Scully S. R., Hardin B. E., Rowell M. W., McGehee M. D. (2007). Polymer based solar cells. Mater. Today.

[cit33] Jankovic V., Yang Michael Y., You J., Dou L., Liu Y., Cheung P. (2013). *et al.*, Active layer-incorporated, spectrally tuned Au/SiO_2_ core/shell nanorod-based light trapping for organic photovoltaics. ACS Nano.

[cit34] Morfa A. J., Rowlen K. L., Reilly III T. H., Romero M. J., van de Lagemaat J. (2008). Plasmon-enhanced solar energy conversion in organic bulk heterojunction photovoltaics. Appl. Phys. Lett..

[cit35] Chen F., Wu J., Lee C., Hong Y., Kuo C., Huang M. H. (2009). Plasmonic-enhanced polymer photovoltaic devices
incorporating solution-processable metal nanoparticles. Appl. Phys. Lett..

[cit36] Shen H., Bienstman P., Maes B. (2009). Plasmonic absorption enhancement in organic solar cells with thin active layers. J. Appl. Phys..

[cit37] Duche D., Torchio P., Escoubas L., Monestier F., Simon J.-J., Flory F., Mathian G. (2009). Improving light absorption in organic solar cells by plasmonic contribution. Sol. Energy Mater. Sol. Cells.

[cit38] Fung D. D. S., Qiao L., Choy W. C. H., Wang C., Sha W. E. I., Xie F., He S. (2011). Optical and electrical properties of efficiency enhanced polymer solar cells with Au nanoparticles in a PEDOT-PSS layer. J. Mater. Chem..

[cit39] Lee J.-Y., Peumans P. (2010). The origin of enhanced optical absorption in solar cells with metal nanoparticles embedded in the active layer. Opt. Express.

[cit40] Spyropoulos G. D., Stylianakis M. M., Stratakis E., Kymakis E. (2012). Organic bulk heterojunction photovoltaic devices with surfactant-free Au nanoparticles embedded in the active layer. Appl. Phys. Lett..

[cit41] Wang D. H., Kim J. K., Lim G.-H., Park K. H., Park O. O., Lim B., Park J. H. (2012). Enhanced light harvesting in bulk heterojunction photovoltaic devices with shape-controlled Ag nanomaterials: Ag nanoparticles *versus* Ag nanoplates. RSC Adv..

[cit42] Wang C. C. D., Choy W. C. H., Duan C., Fung D. D. S., Sha W. E. I., Xie F.-X., Huang F., Cao Y. (2012). Optical and electrical effects of gold nanoparticles in the active layer of polymer solar cells. J. Mater. Chem..

[cit43] Ahn S., Rourke D., Park W. (2016). Plasmonic nanostructures for organic photovoltaic devices. J. Opt..

[cit44] Catchpole K. R., Polman A. (2008). Plasmonic solar cells. Opt. Express.

[cit45] Pillai S., Green M. A. (2010). Plasmonics for photovoltaic applications. Sol. Energy Mater. Sol. Cells.

[cit46] Lance Kelly K., Coronado E., Zhao L. L., Schatz G. C. (2003). The Optical Properties of Metal Nanoparticles: The Influence of Size, Shape, and Dielectric Environment. J. Phys. Chem. B.

[cit47] Jeong S.-H., Choi H., Kim J. Y., Lee T.-W. (2015). Silver-Based Nanoparticles for Surface Plasmon Resonance in Organic Optoelectronics. Part. Part. Syst. Charact..

[cit48] Ma W., Yang C., Gong X., Lee K., Heeger A. J. (2005). Thermally stable, efficient polymer solar cells with nanoscale control of the interpenetrating network morphology. Adv. Funct. Mater..

[cit49] Kim Y., Cook S., Tuladhar S. M., Choulis S. A., Nelson J., Durrant J. R., Bradley D. D. C., Giles M., McCulloch I., Ha C.-S., Ree M. (2006). A strong regioregularity effect in self-organizing conjugated polymer films and high efficiency polythiophene: fullerene solar cells. Nat. Mater..

[cit50] Li W., Furlan A., Hendriks K. H., Wienk M. M., Janssen R. A. J. (2013). Efficient Tandem and Triple-Junction Polymer Solar Cells. J. Am. Chem. Soc..

[cit51] Kim I., Jeong D. S., Seong T., Lee W. S., Lee K.-S. (2012). Plasmonic absorption enhancement in organic solar cells by nano disks in a buffer layer. J. Appl. Phys..

[cit52] Ren W., Zhang G., Wu Y., Ding H., Shen Q., Zhang K., Li J., Pan N., Wang X. (2011). Broadband absorption enhancement achieved by optical layer mediated plasmonic solar cell. Opt. Express.

[cit53] Sefunc M. A., Okyay A. K., Demir H. V. (2011). Plasmonic back contact grating for P3HT:PCBM organic solar cells enabling strong optical absorption increased in all polarizations. Opt. Express.

[cit54] Scharber M. C., Mühlbacher D., Koppe M., Denk P., Waldauf C., Heeger A. J., Brabec C. J. (2006). Design rules for donors in bulk-heterojunction solar cells – Towards 10% energy-conversion efficiency. Adv. Mater..

[cit55] Adebanjo O., Maharjan P. P., Adhikary P., Wang M., Yangc S., Qiao Q. (2013). “Triple junction polymer solar cells”. Energy Environ. Sci..

[cit56] Kim J., Cho K., Kim I., Kim W. M., Lee T. S., Lee K.-S. (2012). Fabrication of Plasmonic Nanodiscs by Photonic Nanojet Lithography. Appl. Phys. Express.

[cit57] Lee S.-W., Lee K.-S., Ahn J., Lee J.-J., Kim M.-G., Shin Y.-B. (2011). Highly Sensitive Biosensing Using Arrays of Plasmonic Au Nanodisks Realized by Nanoimprint Lithography. ACS Nano.

[cit58] Kumar K., Kumawat U. K., Mital R., Dhawan A. (2019). Light trapping plasmonic butterfly-wing-shaped nanostructures for enhanced absorption and efficiency in organic solar cells. J. Opt. Soc. Am. B.

[cit59] Sze S. M. and Ng K. K., Physics of Semiconducting Devices, John Wiley and Sons, 2007

[cit60] Wu J. L., Chen F. C., Hsiao Y. S., Chien F. C., Chen P., Kuo C. H., Huang M. H., Hsu C. S. (2011). Surface plasmonic effects of metallic nanoparticles on the performance of polymer bulk heterojunction solar cells. ACS Nano.

[cit61] Luo Q., Zhang C., Deng X., Zhu H., Li Z., Wang Z., Chen X., Huang S. (2017). Plasmonic Effects of Metallic Nanoparticles on Enhancing Performance of Perovskite Solar Cells. ACS Appl. Mater. Interfaces.

[cit62] Horrer A., Schäfer C., Broch K., Gollmer D. A., Rogalski J., Fulmes J., Zhang D., Meixner A. J., Schreiber F., Kern D. P., Fleischer M. (2013). Parallel fabrication of plasmonic nanocone sensing arrays. Small.

